# NEOGAMES: A Serious Computer Game That Improves Long-Term Knowledge Retention of Neonatal Resuscitation in Undergraduate Medical Students

**DOI:** 10.3389/fped.2021.645776

**Published:** 2021-04-21

**Authors:** Liyuan Hu, Lan Zhang, Rong Yin, Zhihua Li, Jianqing Shen, Hui Tan, Jingyan Wu, Wenhao Zhou

**Affiliations:** ^1^Department of Education and Training, Children's Hospital of Fudan University, Shanghai, China; ^2^Department of Neonatology, Children's Hospital of Fudan University, Shanghai, China

**Keywords:** serious games, neonatal resuscitation, knowledge retention, medical education, simulation-based education

## Abstract

**Background:** Serious games are potential alternatives for supplementing traditional simulation-based education for neonatal resuscitation training. However, evidence regarding the benefits of using serious games to improve long-term knowledge retention of neonatal resuscitation in undergraduate medical students is lacking.

**Objective:** We designed a serious computer game “NEOGAMES” to train undergraduate medical students in neonatal resuscitation in a cost-friendly and accessible way and to examine whether serious game-based training improves long-term knowledge retention in medical students.

**Methods:** “NEOGAMES” consists of a screen with images of an incubator, a baby, visual objects, anatomy, action cards, monitors, real-time feedback, and emotional components. Undergraduate medical students from Shanghai Medical College of Fudan University were invited to participate and were allocated to a game group or a control group. Participants in the game group played the game before the training. All the participants completed three written tests, pre- and post-training knowledge tests and a follow-up test after 6 months.

**Results:** Eighty-one medical students participated in the study. The student demographic characteristics of the groups were comparable, including sex, age, and grade point average (GPA). Significant short-term knowledge improvement was noticed only for male students in the game group based on their 5.2-point higher test scores than those of the controls (*p* = 0.006). However, long-term knowledge improvement at 6 months was identified for both male and female students in the game group, with test scores 21.8 and 20 points higher, respectively, than those of the controls (*P* < 0.001). The long-term knowledge retention in the game group was almost 3 times higher than that in the control group.

**Conclusions:** Long-term knowledge retention was nearly 3 times higher for the game group than for the control group. The improvement in knowledge supports the use of serious games for undergraduate medical education.

## Introduction

Simulation is beneficial for improving performance initially after training and is a time-effective method that has been used globally (1); however, cognitive and technical skills significantly deteriorate within months (2), and deficiencies in non-technical skills remain common (3). Moreover, the current simulation-based medical education approach is expensive and time intensive and requires numerous resources and trained instructors. Therefore, other training methods for improving knowledge retention and decision-making are emerging (4).

Serious games are potential alternatives for supplementing traditional simulation-based education (SBE) for neonatal resuscitation training and are able to improve working memory, decision-making and teamwork performance at low cost (5). They are increasingly incorporated into medical school curricula to reinforce theoretical and practical learning (5).

Short-term knowledge improvement was shown by the Neonatology Game 4.2-points higher test scores than those of a control in medical students (6). Ghoman et al. trained 50 health care providers (HCPs) with the RETAIN digital simulation game and their short- and long-term knowledge retention was evaluated (7). Proportion of correct performance significantly increased immediately after training (pre- vs. post-test, 42% vs. 78%, respectively), and was retained 2-months (follow-up post-test I, 70%) and 5-months after training (follow-up post-test II, 80%). However, this study was conducted in a population of experienced NRP-trained healthcare professionals.

So far, no research has examined the use of a serious game to improve the long-term knowledge retention of neonatal resuscitation for undergraduate medical students, thus highlighting a gap in the literature. We designed the serious computer game NEOGAMES (Neonatal Resuscitation Simulation Game Designed for Medical Students) and introduced it into formal SBE for neonatal resuscitation training in medical students. We conducted this study with the aims of bridging this gap by providing evidence for the use of a serious game for undergraduate medical education. We hypothesized that the implementation of the serious computer game NEOGAMES would effectively improve undergraduate medical students' short- and long-term knowledge retention of neonatal resuscitation.

## Materials and Methods

### Ethical Approval

This study was conducted in accordance with the Declaration of Helsinki (8) and was approved by the Research Ethics Committee of the Children's Hospital of Fudan University (No. 2020300). Informed consent was obtained from all the participants.

### Developing the NEOGAMES Computer Game

First, nine neonatologists from the Department of Neonatology at Children's Hospital of Fudan University were interviewed about what knowledge and skills were essential for neonatal resuscitation but difficult to acquire for medical students. The responses were (i) knowledge of indications to initiate resuscitation, (ii) knowledge of how to choose appropriate equipment and medications, and (iii) intubation skills.

Next, the NEOGAMES computer game (http://www.ilab-x.com/details?id=2785&isView=true, Children's Hospital of Fudan University, Shanghai, China) was designed based on the interview responses. This game obtained a certificate of Registration of Computer Software Rights by the National Copyright Office of the People's Republic of China (No: 2019SR1005248).

**Figure 1:**
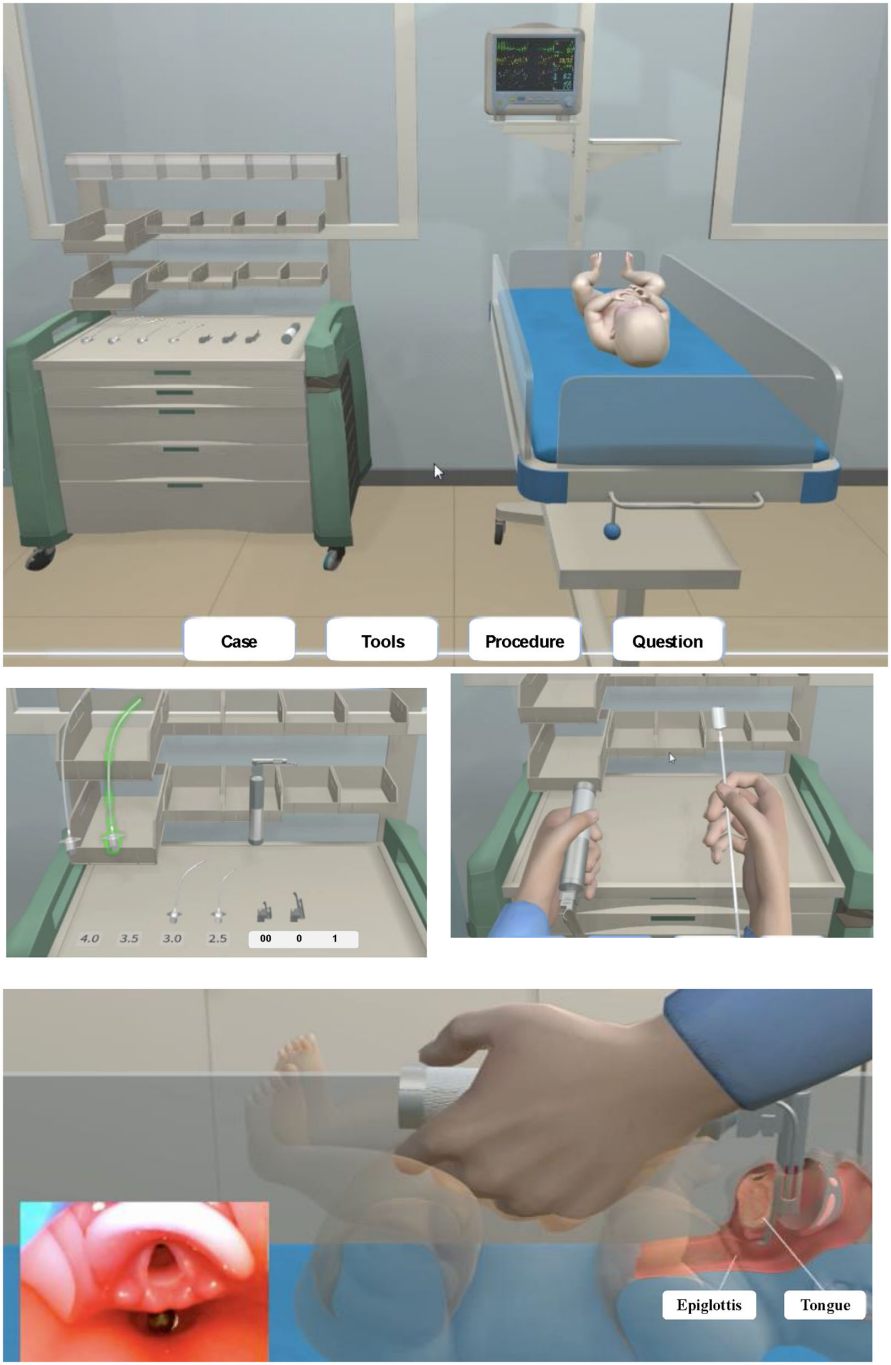
The NEOGAMES (Neonatal Resuscitation Simulation Game Designed for Medical Students) neonatal resuscitation computer game.

The game can be played individually on the web with free access. One play through the game takes ~10 min. In the game, a trainee is presented with an evidence-based scenario derived from real-life delivery room resuscitation instances at the Children's Hospital of Fudan University, Shanghai, China. The scenario occurs in a labor and delivery room where a term cyanotic neonate with heartbeat from 50 to 60 beats per minute has just been delivered. The trainee can check heart rate, oxygen saturation and blood pressure and must perform resuscitations using equipment and supplies and make decisions according to the algorithm of the 2015 guidelines of Neonatal Resuscitation Program (9) and vital signs. The visual objects representing tools and equipment are placed on the resuscitation cart. The game also includes 7 question cards (e.g., start intubation, choose the appropriate tube and blade, find the correct anatomy for intubation, decide the appropriate depth for the endotracheal tube) with real-time feedback after each decision. Resuscitation occurs only when the trainee makes the right decision or corrects the wrong decision for each action. At the end of the game, the critically ill neonate is resuscitated with a heartbeat increase to 150 beats per minute, skin color change from blue to pink and congratulations given to the trainee for saving the neonate's life. The trainee is presented with the last question card to test short-term knowledge retention after one scenario. A total of the overall points at the end of the game is displayed based on the performance of all the actions (Figure 1).

### Study Design

**Figure 2:**
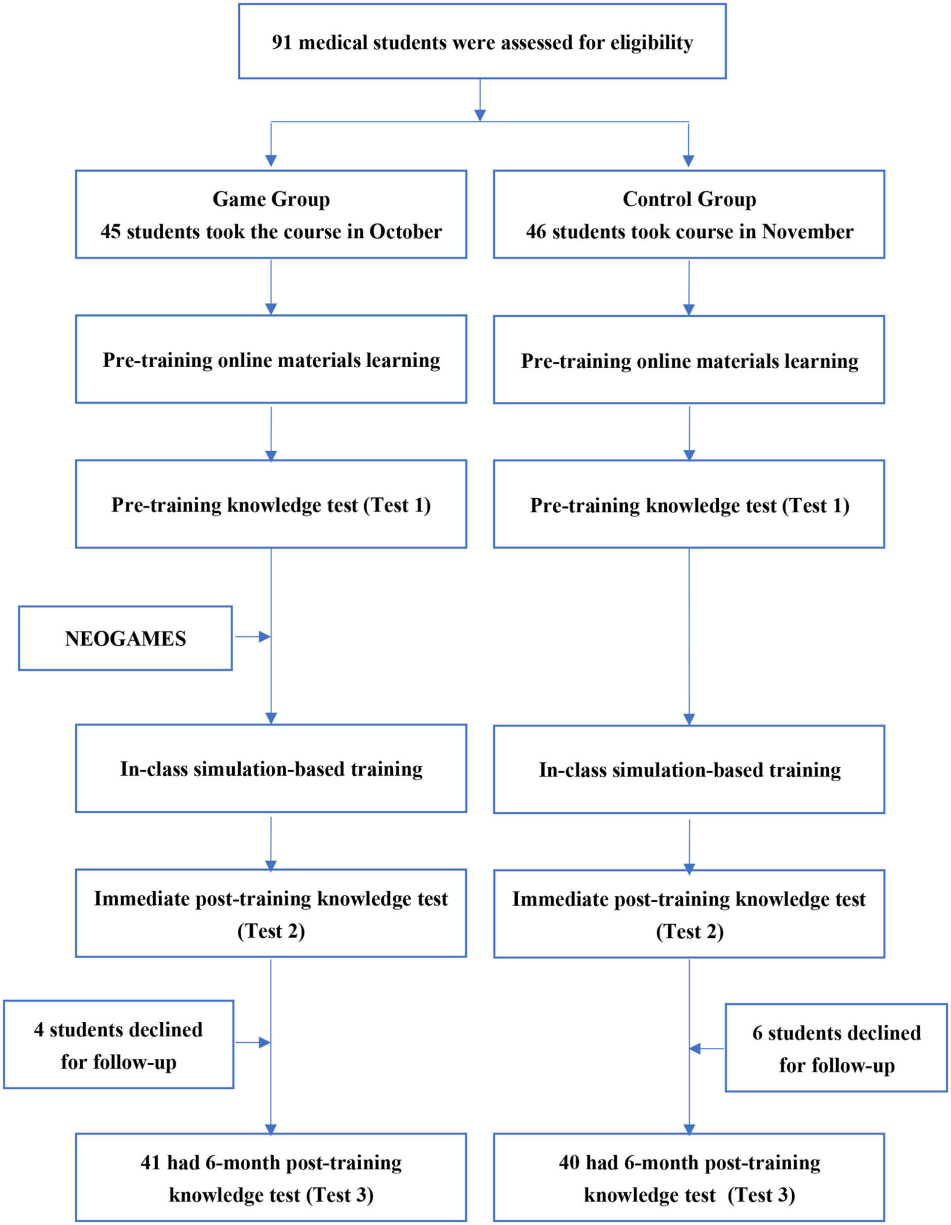
Participant flow and study design.

The study design was divided into three steps (Figure 2).

Step 1: Pre-training baseline knowledge test. Before the training, all the students were asked to study one school hour of same online materials related to the course. Then, they completed an online baseline knowledge test about resuscitation (Test 1, included questions about the indications for resuscitation, medication, intubation, etc.) at home that they could consulted their study material while taking the test. For the game group, the participants were asked to play the free online NEOGAMES computer game before attending the in-class simulation training (Figure 2).

Step 2: Simulation-based training and post-training test. The students underwent simulation-based training in the simulation center at the Children's Hospital of Fudan University and completed a second knowledge test (Test 2) immediately after the class. The training course lasted for 2 school hours. The students received skill training for intubation, chest compression, medication and indications for different resuscitation steps. Four neonatologists skilled in simulation-based education (SBE) were trained together before the study. They were responsible for the SBE training and for giving students real-time feedback in class (Figure 2).

Step 3: Follow-up knowledge test at 6 months after the training. The students were asked to finish a knowledge assessment regarding resuscitation 6 months after the training (Test 3, included questions about the indications for resuscitation, medication, intubation, etc.) (Figure 2). In between the 6-months, the game group was not allowed to play the game.

The maximum scores for NEOGAMES, pre-test, post-test, and follow-up test were all 100 points.

### Subjects Selection

A prospective cohort study was carried out among the fourth and fifth years of undergraduate medical students from Shanghai Medical College of Fudan University who took a neonatal resuscitation training course at the Children's Hospital of Fudan University between October 1st and December 31st in 2019. All the undergraduate medical students didn't have any previous experience with neonatal resuscitation or training before participating in the study. The allocation was not randomized. The students who took the course in October were allocated to the game group with access to the online NEOGAMES. Those who took the course in November were allocated to the control group without access to NEOGAMES. Students who declined to participate in the follow-up evaluation test were excluded from the final analysis (Figure 2).

### Data Collection and Statistical Analysis

Continuous variables are expressed as the means ± standard deviations. Categorical variables are presented as percentages. Average pre-test, post-test, and follow-up scores were compared using a paired *t*-test. Univariate analyses of categorical data were conducted with chi-square and Fisher exact-tests. Differences were considered statistically significant with a two-tailed *p*-value < 0.05. The analysis was performed using IBM SPSS 25.0.

## Results

### Participant Characteristics

A total of 91 undergraduate medical students were eligible for the study, and all of them agreed to participate (100% response rate). Forty-five students who took the course in October were allocated to the game group. Those who took the course in November were allocated to the control group. In total, 4 students in the game group and 6 students in the control group declined to participate in the 6-month follow-up evaluation, leaving 41 and 40 participants in the game and control groups, respectively (Figure 2), resulting in a follow-up response rate of 89.0%.

Finally, a total of 39 male and 42 female participants with a mean age of 22.2 ± 0.6 years were enrolled in the final analysis. The student demographic characteristics, including sex, age, and grade point average (GPA) for the previous semester were similar across groups. The participant characteristics are provided in Table 1.

**Table 1:** Baseline characteristics of the enrolled participants^*^.

**Characteristics^**‡**^**	**Game group (*N* = 41)**	**Control group (*N* = 40)**	***P*-value**
Age—year	22.1 ± 0.5	22.3 ± 0.6	0.063
Female sex —no (%)	24 (58.5)	18 (45.0)	0.223
GPA	3.0 ± 0.5	3.1 ± 0.5	0.125
Female	3.1 ± 0.4^**^	3.2 ± 0.5^&^	0.234
Male	2.8 ± 0.5^**^	3.0 ± 0.5^&^	0.154

* *Plus–minus values are means ± SD*.

‡ *There was no significant difference in age, sex or GPA between the game and control groups*.

** *There was no significant difference in GPA between the male and female (P = 0.058) in the game group*.

& *There was no significant difference in GPA between the male and female (P = 0.117) in the control group*.

### Knowledge Retention

The mean scores of the pre-training baseline knowledge test for the game and control groups remained similar (*P* = 0.281), 93.1 ± 8.2, and 91.2 ± 6.9, respectively.

In the control group, SBE was essential for improving short-term knowledge by 4.2 points (*P* = 0.007). There was a significant short-term knowledge improvement, with a 3.3-point higher test score than that of the control (*p* = 0.006). The mean scores of a follow-up knowledge test at 6 months in the game group were 23.9 points higher than those in the control group (*P* < 0.001). Participants who underwent NEOGAMES computer game training experienced increased short- and long-term knowledge compared to participants in the control group.

**Figure 3:**
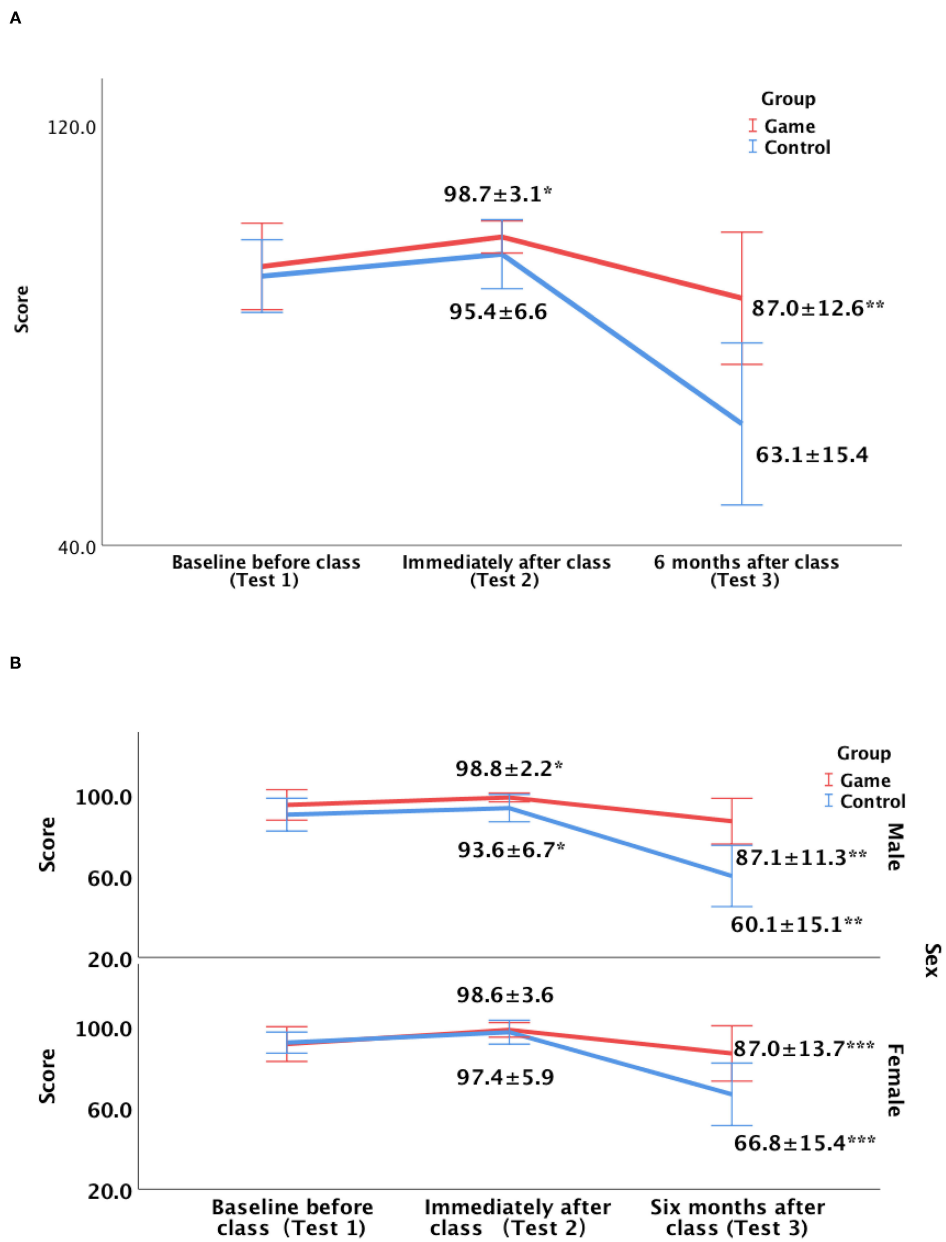
Trends of knowledge retention at 6 months after class for the game and control groups. Red and blue lines indicate the trend of the mean test scores of the game and control groups. **(A)** The mean test scores were best immediately after class but decreased over time, by 11.7 points in the game group and 32.3 points in the control group at follow-up, respectively. * and ** indicate that the mean post-test scores immediately and 6 months after class were significantly higher in the game group than in the control group, with *p*-values of 0.006 and <0.001, respectively. The intra-group performance longitudinally over time differed significantly, with p values of <0.001 in both game and control groups. **(B)** The upper and lower panels represent the trend of male and female students, respectively. * indicates that the mean test scores immediately after class (test 2) were significantly different between the game and control groups for only the male students (*P* = 0.003). ** and *** indicate that both the male and female students from the game group had significant higher mean scores at 6-month follow-up (test 3) than those from the control group (*P* < 0.001).

Figure 3A shows the trends of knowledge retention after training. The mean test scores were best immediately after SBE but decreased over time, by 11.7 points in the game group and 32.3 points in the control group at the 6-month follow-up. However, the knowledge retention in the game group was almost 3 times higher than that in the control group.

To explore the impact of NEOGAMES in improving short- and long-term knowledge retention by sex, further subgroup analysis was carried out according (Figure 3B). Significant short-term knowledge improvement was noticed only for the male students in the game group, with 5.2-point higher test scores than those of the controls (*p* = 0.006). However, long-term knowledge improvement at 6 months after SBE was identified for both male and female students in the game group, with 21.8- and 20-point higher test scores than those of the controls, respectively (*P* < 0.001).

## Discussion

Simulation is beneficial for improving performance initially after training, but the skills significantly deteriorate within months (1, 2). In our study, we found similar results: simulation was beneficial for improving performance initially after training (91.2 ± 6.9 vs. 95.4 ± 6.6, *p* = 0.007) but significantly deteriorated after 6 months (95.4 ± 6.6 vs. 63.1 ± 15.4, *p* < 0.001).

We found that the addition of a serious game enhanced the male students' short-term knowledge retention, with scores 5.2 points higher than those of the controls (*p* = 0.006). The underlying causes for this finding remain unknown.

Games have been found to be a very effective method for reviewing material, reinforcing facts and enhancing motivation to promote the learning outcomes of adult learners (10, 11). Cutumisu et al. found that 30 health care providers who played the RETAIN board game (RETAIN Labs Medical, Edmonton, Alberta, Canada) had a 12% increase in knowledge retention; however, the sample size was small, and long-term follow-up data were not available (4). Swiderska et al. found that students who played the Neonatology Game (University of Glasgow, Glasgow, UK) had a mean post-test score that was 4.2 points higher than that of the control group. However, there was neither a pre-test for baseline comparison nor a follow-up to explore long-term knowledge retention (6).

Ghoman et al. trained 50 health care providers (HCPs) with the RETAIN digital simulation and found that the digital simulation effectively improved, maintained, and helped transfer HCPs' neonatal resuscitation knowledge over time (7). However, no research has examined the efficacy of a serious game to improve medical students' long-term knowledge retention of neonatal resuscitation. Our study was the first to investigate the efficacy of a serious game on long-term knowledge improvement at 6 months in medical students. We found that the male and female students in the game group had 21.8 and 20 points higher test scores than the controls, respectively (*P* < 0.001), and these scores were almost 3 times higher than those of the control group. This promising finding was able to bridge the gap and provide evidence for the use of a serious game for undergraduate medical education in addition to SBE.

Neonatal resuscitation is a highly stressful medical emergency requiring quick and correct decisions under elevated time pressure. Deficiencies in non-technical skills rather than technical skills are the cause of the majority of fatal errors and poor patient outcomes. Therefore, it is important to improve long-term working memory, decision-making and teamwork performance according to neonatal resuscitation guidelines (12–14).

Thus, NEOGAMES was designed to improve long-term working memory and decision-making. It is widely available with minimal setup and no cost, is flexible and easily accessible to trainees *via* the internet and may be able to provide an attractive and intrinsically motiving learning experience through its real-time feedback, competition, emotional components (e.g., heartbeat and cyanosis) and autonomy. These characteristics of NEOGAMES might be one of the explanations for the positive findings.

Although medical education may benefit from incorporating technology during simulation training (15), using an unguided web-based game alone may not be effective in facilitating retention of knowledge and technical skills in neonatal resuscitation (16). Thus, we added NEOGAMES to formal SBE for neonatal resuscitation in medical students to prevent bias. The participants in the game group were asked to play the online free NEOGAMES computer game before attending the in-class simulation training. The combination of a serious game and SBE might be another explanation for the promising results.

## Limitations

The limitations of NEOGAMES include accessibility (e.g., access to a computer or internet), high cost of initial development and requirement for consistent updating. The findings from this study should be interpreted in light of several limitations. First, all the participants were undergraduate medical students from Fudan University in Shanghai and were not randomized, which could result in selection bias. Second, the sample was not large, and future research should enroll more students to verify these findings. Third, learning outcomes include knowledge, skills, and attitude, and future studies are required to determine the efficacy of long-term skill retention and important clinical outcomes in newborn infants. A major strength is this paper's examination of both short-term and long-term knowledge retention, as well as evaluation of all participants' baseline knowledge. Fourth, students in the NEOGAMES group simply benefitted from additional study time, which may contribute to improved performance in 6 months. Future study should have the control group study additional non-game materials, so that both the control and intervention group spend the same amount of time studying neonatal resuscitation.

## Conclusions

The use of the specifically developed educational neonatal resuscitation game NEOGAMES could facilitate learning and promote short-term and long-term knowledge retention in medical students. Moreover, NEOGAMES might be used as a formative or summative assessment and as a pre-instructional assessment tool to identify medical students' strengths and weaknesses. Future studies are warranted to confirm these findings.

## Data Availability Statement

The original contributions presented in the study are included in the article/supplementary material, further inquiries can be directed to the corresponding authors.

## Ethics Statement

The studies involving human participants were reviewed and approved by the Research Ethics Committee of the Children's Hospital of Fudan University (No. 2020300). The patients/participants provided their written informed consent to participate in this study.

## Author Contributions

JW and WZ: conception and design. HT and JS: administrative support. LH and WZ: provision of study materials or patients. ZL, RY, and LZ: collection and assembly of data. All authors: data analysis and interpretation, manuscript writing, and final approval of manuscript.

## Conflict of Interest

The authors declare that the research was conducted in the absence of any commercial or financial relationships that could be construed as a potential conflict of interest.

## References

[1] Kaczorowski J Levitt C Hammond M. Retention of neonatal resuscitation skills and knowledge: a randomized controlled trial. Fam Med. (1998) 30:705–11. 9827341

[2] Mileder LP Urlesberger B Szyld EG Roehr CC Schmölzer GM. Simulation-based neonatal and infant resuscitation teaching: a systematic review of randomized controlled trials. Klin Padiatr. (2014) 226:259–67. 10.1055/s-0034-1372621 25153910

[3] Niermeyer S. From the neonatal resuscitation program to helping babies breathe: global impact of educational programs in neonatal resuscitation. Semin Fetal Neonatal Med. (2015) 20:300–8. 10.1016/j.siny.2015.06.005 26265602

[4] Cutumisu M Patel SD Brown MRG Fray C von Hauff P Jeffery T. RETAIN: a board game that improves neonatal resuscitation knowledge retention. Front Pediatr. (2019) 7:13. 10.3389/fped.2019.00013 30766862 PMC6365420

[5] Ghoman SK Patel SD Cutumisu M von Hauff P Jeffery T Brown MR. Serious games, a game changer in teaching neonatal resuscitation? A review. Arch Dis Child Fetal Neonatal Ed. (2020) 105:98–107. 10.1136/archdischild-2019-317011 31256010 PMC6951231

[6] Swiderska N Thomason E Hart A Shaw BN. Randomised controlled trial of the use of an educational board game in neonatology. Med Teach. (2013) 35:413–5. 10.3109/0142159X.2013.769679 23444884

[7] Ghoman SK Cutumisu M Schmölzer GM. Digital simulation improves, maintains, and helps transfer health-care providers' neonatal resuscitation knowledge. Front Pediatr. (2021) 8:599638. 10.3389/fped.2020.599638 33537263 PMC7848194

[8] World Medical Association. World medical association declaration of Helsinki: ethical principles for medical research involving human subjects. JAMA. (2013) 310:2191–4. 10.1001/jama.2013.281053 24141714

[9] Wyckoff MH Aziz K Escobedo MB Kapadia VS Kattwinkel J Perlman JM. Part 13: neonatal resuscitation: 2015 American Heart Association guidelines update for cardiopulmonary resuscitation and emergency cardiovascular care. Circulation. (2015) 132(Suppl. 2):S543–60. 10.1161/CIR.0000000000000267 26473001

[10] Rutledge C Walsh CM Swinger N Auerbach M Castro D Dewan M. Gamification in action: theoretical and practical considerations for medical educators. Acad Med. (2018) 93:1014–20. 10.1097/ACM.0000000000002183 29465450

[11] Bigdeli S Kaufman D. Digital games in medical education: key terms, concepts, and definitions. Med J Islam Repub Iran. (2017) 31:52. 10.14196/mjiri.31.52 29445681 PMC5804455

[12] Langhan TS Rigby IJ Walker IW Howes D Donnon T Lord JA. Simulation-based training in critical resuscitation procedures improves residents' competence. CJEM. (2009) 11:535–9. 10.1017/S1481803500011805 19922713

[13] Hunziker S Pagani S Fasler K Tschan F Semmer NK Marsch S. Impact of a stress coping strategy on perceived stress levels and performance during a simulated cardiopulmonary resuscitation: a randomized controlled trial. BMC Emerg Med. (2013) 13:8. 10.1186/1471-227X-13-8 23607331 PMC3640892

[14] Müller MP Hänsel M Fichtner A Hardt F Weber S Kirschbaum C. Excellence in performance and stress reduction during two different full-scale simulator training courses: a pilot study. Resuscitation. (2009) 80:919–24. 10.1016/j.resuscitation.2009.04.027 19467753

[15] Lu C Ghoman SK Cutumisu M Schmölzer GM. Unsupervised machine learning algorithms examine healthcare providers' perceptions and longitudinal performance in a digital neonatal resuscitation simulator. Front Pediatr. (2020) 8:544. 10.3389/fped.2020.00544 33042905 PMC7518390

[16] Yeo CL Ho SKY Tagamolila VC Arunachalam S Bharadwaj SS Poon WB. Use of web-based game in neonatal resuscitation—is it effective? BMC Med Educ. (2020) 20:170. 10.1186/s12909-020-02078-5 32456704 PMC7249390

